# Effects of endocrine disorders on maxillary and mandibular growth in Colombian children and adolescents: a cross-sectional study

**DOI:** 10.1007/s40368-023-00850-x

**Published:** 2023-11-24

**Authors:** J. M. Alfaro, R. Manrique, A. Santamaría, E. Álvarez, C. Manes, M. Jiménez

**Affiliations:** 1 Pediatric Endocrinologist, Pediatric Research Group, Medical School, CES Clinic, Medellín, Colombia; 2 Epidemiology and Biostatistics Research Group, CES University, Medellín, Colombia; 3 LPH Research Group, Dental School, CES University, Medellín, Colombia; 4 Head and Neck Bioengineering Research Group, Dental School, CES University, Medellín, Colombia; 5 Master’s Degree in Dental Sciences, CES University, Medellín, Colombia

**Keywords:** Thyroid disorders, Obesity, Growth hormone deficiency, Anthropometry

## Abstract

**Objective:**

To establish the influence of overweight/obesity, medicated hypothyroidism, and medicated non-syndromic hypogrowth on maxillary and mandibular growth.

**Materials and methods:**

The relation between 10 craniofacial anthropometric measurements and hypothyroidism (*n* = 216), overweight/obesity (*n* = 108), and non-syndromic hypogrowth (*n* = 250) were evaluated in patients aged 1–19 years and a control group of healthy patients (*n* = 587). A subgroup analysis was performed at the peak growth in all groups.

**Results:**

Patients with overweight/obesity and hypothyroidism showed increased craniofacial growth, while hypogrowth patients showed differences in zygomatic width and nasal base growth. Females with hypothyroidism and non-syndromic hypogrowth showed decreased head circumference at peak growth. Several anthropometric measurements were increased in patients with overweight/obesity, including head circumference. When all age groups were analyzed, overweight/obese and hypothyroidism patients showed increased zygomatic width while decreased hypogrowth. Overall, most craniofacial anthropometric measurements in overweight/obese patients were increased. Finally, the peak growth in males with hypothyroidism and subjects with non-syndromic hypogrowth was delayed compared to the control group (*p* < 0.05).

**Conclusions:**

Children and adolescents with overweight/obesity and endocrine disorders showed alterations in craniofacial growth. Clinicians must be aware that the growth peak in these patients may be delayed when planning maxillary and mandibular orthopedic treatment.

## Introduction

General somatic growth, specifically craniofacial growth, depends on interacting processes that transform humans from birth to adulthood (Gorstein et al. 1994). Craniofacial growth is characterized by marked changes in speed, size, shape, and function attributed to genetic, hormonal, and environmental factors. For example, hypothyroid status, obesity, and growth hormone deficiency can alter children’s growth and development, affecting the harmonious relationship between different facial structures (Nahhas et al. 2014). Such conditions continue to increase in several populations, including Latin America. In Colombia, the prevalence of hypothyroidism (referencia de la Asociación Colombiana de Endocrinología, Diabetes y Metabolismo), overweight, and obesity (referencia del Ministerio de Salud) is 9.9%, 37.7%, and 18.7%, respectively. In addition, the prevalence of growth hormone deficiency is 10.8% in children < 5 years, 7.4% in children between 5 and 12, and 9.7% in children between 13 and 17 (referencia del Ministerio de Salud).

Hypothyroidism can affect cardiovascular, neurological, gastrointestinal, and metabolic functions (Wassner 2017). In craniofacial structures, delayed tooth eruption, enamel hypoplasia, micrognathism, retrognathism, anterior open bite, and tooth impaction have been observed (Vuccic et al. 2017). Overweight and obesity can alter bone metabolism, stimulate bone growth, inhibit bone remodeling, and increase the risk of musculoskeletal injuries (Dimitri 2019). Craniofacially, they can induce prognathism (Danze et al. 2021), increased facial height (Cuccia et al. 2007), early tooth eruption (Saloom et al. 2017), and periodontitis (Zhu et al. 2017). Growth hormone deficiency decreases growth velocity, leading to an immature facial appearance, short stature, hair deficiency, and delayed puberty (Ogilvy-Stuart and Shalet 1992). It also can lead to maxillary hypoplasia and mandibular retrognathism (Atanassio 2017), anterior open bite, facial hyperdivergence (Buschang and Hinton 2005), decreased anterior facial height, and short mandibular ramus (Oliveira-Neto et al. 2011). These disorders cause changes in craniofacial growth and development structures, including the maxilla and mandible, however, there is not enough scientific evidence of this association. Thus, it is crucial for clinicians to understand the impact these conditions can have on craniofacial development and peak growth.

Therefore, this study aimed to investigate the influence of medicated hypothyroidism, overweight/obesity, and medicated non-syndromic hypogrowth on maxillary and mandibular growth in children and adolescents, using anthropometric measurements.

## Materials and methods

This quantitative, cross-sectional analytical study was conducted on 1508 children and adolescents between 1 and 19 years. The subjects were divided into four age groups: 1–4, 5–9, 10–14, and 15–19 years. Of these, 216 had a clinical diagnosis of hypothyroidism, 108 were diagnosed with overweight or obesity, 250 had a non-syndromic hypogrowth, and 587 were systemically healthy and regarded as the control group. Since this was a retrospective study, the following eligibility criteria were used for each of the study groups:

- Overweight: a person between 5 and 18 years with a body mass index (BMI) between + 1 standard deviation (SD) (84.1%) and + 2 SD (97.7%), according to the Colombian growth charts.
- Obesity: a person between 5 and 18 years with a body mass index (BMI) above + 2 SD (97.7%), according to the Colombian growth charts.
- Hypothyroidism: low serum T_4_ and elevated serum TSH, accompanied or not goiter and other clinical manifestations such as delayed growth and development, intolerance to cold, bradycardia, cold skin, constipation, muffled heart sounds, and tendon reflexes with low relaxation.
- Non-syndromic hypogrowth: a person with a height value less than − 2 SD (2.3%), according to the Colombian growth charts.
- Healthy: a person between 5 and 18 years who, in successive follow-ups at the Pediatric Endocrinology Outpatient Clinic, did not show alterations in growth, development, or pubertal maturation.

Patients with syndromic hypogrowth or syndromes that might affect craniofacial morphology were not included, nor were children who did not consent or their parents to participate in the study.

Data were collected from the medical records of the Pediatric Endocrinology Outpatient Service at CES Clinic, Medellin, Colombia, between 2014 and 2021, prior participant’s assent, parental’s informed consent, and authorization from the institution. The study was approved by the CES University’s Institutional Ethics Review Board (act 178 of October 28, 2021).

**Fig. 1:**
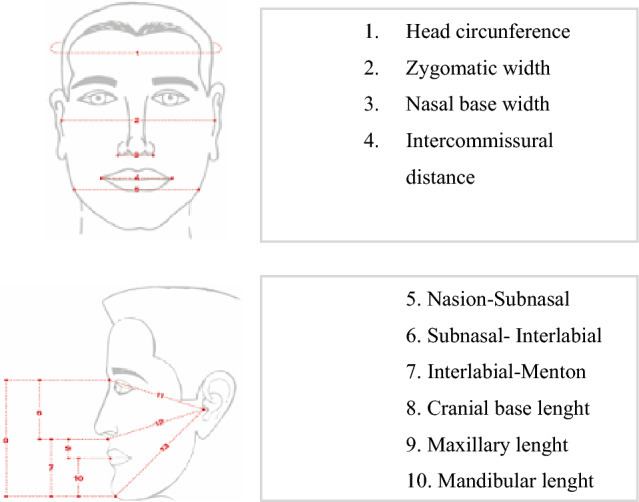
Anthropometric measurement (Alfaro 2014)

**Fig. 2:**
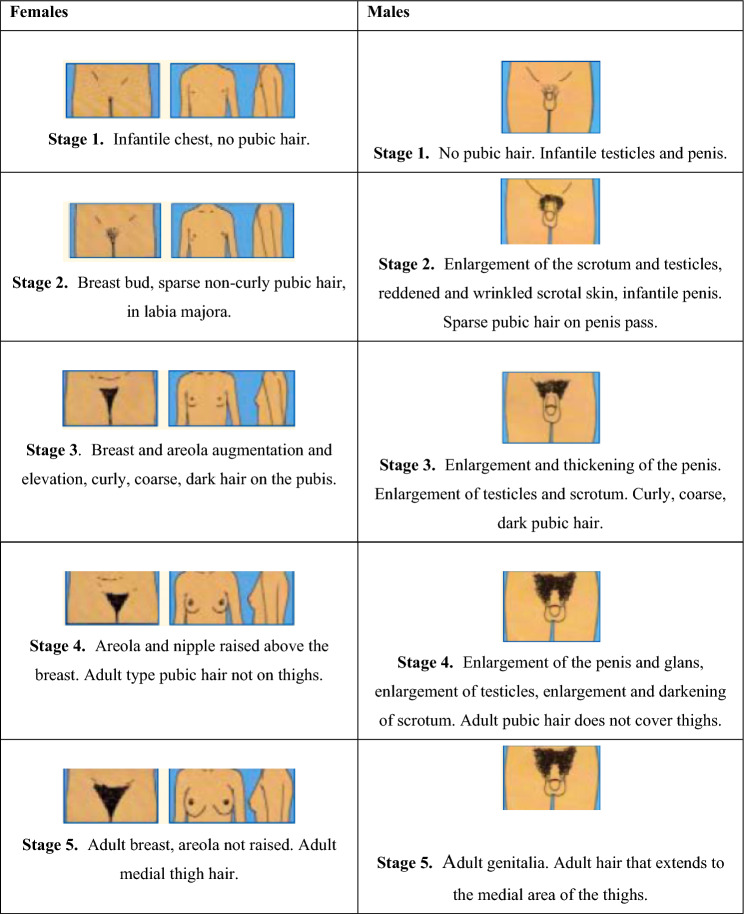
Tanner scale according to gender (Marshall and Tanner 1969)

Patient weight was recorded in grams using a SECA 803 electronic scale and height with a wall measuring rod that complies with Colombian regulations (Resolution 2465 of 2016). Bone maturation, as an indicator of skeletal age, was calculated using the Greulich and Pyle method (Greulich and Pyle 1950). Hypothyroidism was evaluated by measuring TSH and T_4_ hormones with an 8-h fasting blood test. Hypothyroidism was diagnosed when TSH > 6 mU/L or T4 < 0.8 mU/L (based on the Latin American Association of Endocrinology). Obesity was classified at two moments between birth and 5 years. Weight was evaluated when height was more than 3 standard deviations (SD) above the median established for childhood growth standards based on the WHO Growth Chart tables between 5 and 19 years old. BMI was assessed for age and gender with more than 2 standard deviations (SD) above the established median according to the same growth charts. Hypogrowth was diagnosed when height was 2 standard deviations (SD) below the median in the same growth chart (Mercedes 2006). Anthropometric measurements were taken by a single operator using a Mitutoyo reference 530–114 caliper with an error limit of 0.05 mm/m. The operator was previously standardized in the location of points with an experienced endocrinologist with an average intra-observer reliability of 0.97, according to the intraclass correlation coefficient (ICC). Measurements were made at three different times until obtaining an ICC value ≥ 0.95 in every individual. Likewise, a new standardization was performed every time a new group of patients was available with a pilot test of at least 20 readings. Figure 1 shows the reference points for each anthropometric distance measured (Vásquez 2014). Finally, the Tanner stages were used to determine pubertal development (Marshall and Tanner 1970; Molina 2009) (Fig. 2).

### Statistical analysis

To determine the relationship between endocrine disorders and craniofacial development in children and adolescents, a non-probabilistic sample was formed by concurrence based on the medical records of patients who attended the Pediatric Endocrinology Service at CES Clinic in Medellin, Colombia.

A descriptive analysis was conducted in tables and figures for the categorical variables, while quantitative variables such as summary, central tendency, dispersion, and position measures were estimated. The normal distribution of such variables was assessed with the Shapiro–Wilks and Asymmetry and Kurtosis tests with statistical significance set at *p* < 0.05. Given that none of the quantitative variables related to facial anthropometric measurements and somatic growth presented a normal distribution, the comparisons in the bivariate analysis were made using the Mann–Whitney *U* test.

## Results

The analyzed sample consisted of 1506 patients (females, *n* = 765). No information on age and gender was registered from two participants (0.13%) and were therefore not included in the analysis. The age groups with the highest number of patients were 10–14 years (366 males and 352 females) and 5–9 years (179 males and 280 females) (Table 1).

**Table 1:** Age group distribution according to gender

Age (years)	Gender	
	Male	Female
	*n* (%)	*n* (%)
1–4	44 (5.9)	49 (6.4)
5–9	179 (24.2)	280 (36.6)
10–14	366 (49.4)	352 (46.0)
15–19	152 (20.5)	84 (11.0)
Total	741	765

*n* sample size

As shown in Table 2, 921 (61%) subjects presented some type of disorder, and 587 (38.9%) were healthy. No significant differences in the proportion of males and females were found in healthy individuals and those with obesity. In contrast, there was a higher proportion of girls in the medicated hypothyroidism group (*p* = 0.002) and a higher proportion of boys in the non-syndromic hypogrowth group compared to girls (*p* < 0.000). Other comorbidities found in this population were diabetes, dyslipidemia, thyroiditis, teenage pregnancy and hyperthyroidism, which were not considered for the analysis due to the difference in proportions compared with other disorders.

**Table 2:** Diseases in the study population according to gender

Disorder	Gender			*p*-value
	Male	Female	Total	
	*n* (%)	*n* (%)	*n* (%)	
Healthy	285 (38.5)	302 (39.5)	587 (38.9)	0.685
Medicated hypothyroidism	85 (11.5)	131 (17.1)	216 (14.3)	0.002^a^
Overweight/obesity	50 (6.8)	58 (7.6)	108 (7.2)	0.533
Non-syndromic hypogrowth	174 (23.5)	76 (9.9)	250 (16.6)	0.000^a^
Other endocrine disorders	147 (19.8)	198 (25.9)	347 (23.0)	0.005^a^
Total	741 (100)	765 (100)	1.508 (100)	

*n* sample size

^a^*p*-value calculated with the non-parametric Mann–Whitney *U* test

**Fig. 3:**
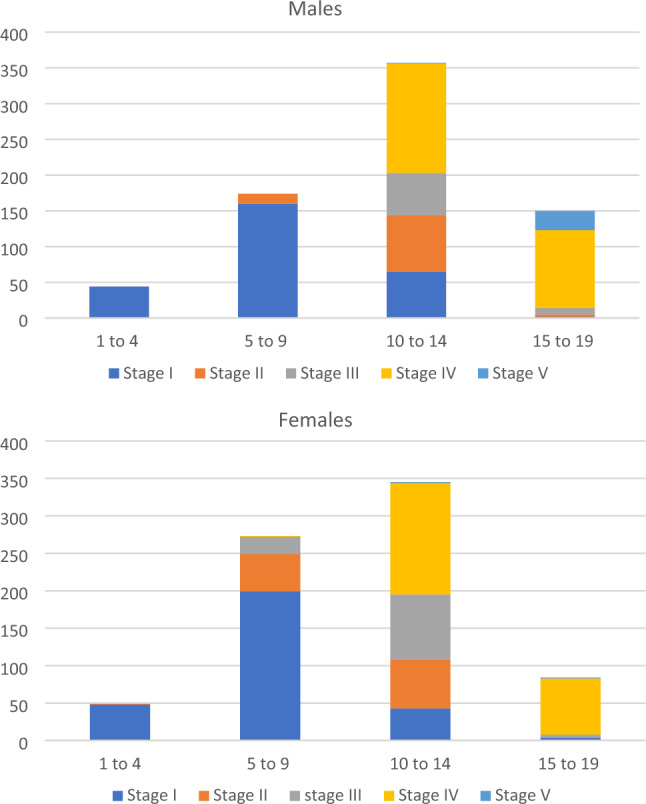
Tanner scores according to chronological age and gender

No significant differences in Tanner scores were observed between boys and girls in the 1–4 years age group. However, in the 5–9 years age group, most boys presented a Tanner I stage, while in girls, an increased proportion of stages II, III, and IV was found. In the 10–14 age group, a similar gender distribution was observed, while in the 15–19 age group, both Tanner IV and V scores represented the highest proportion (Fig. 3).

There were significant differences in anthropometric measurements between healthy subjects and those with hypothyroidism, obesity/overweight, and non-syndromic hypogrowth (Table 3). Although the mean and standard deviation are shown, groups were compared using a Mann–Whitney *U* non-parametric test, given that none of the measurements followed a normal distribution. Children with concomitant hypothyroidism and overweight/obesity presented significantly different measurements, i.e., head circumference, zygomatic width, nasal base width, intercommissural distance, cranial base length, maxillary length, and mandibular length compared with healthy individuals. In addition, subnasal-interlabial and interlabial-menton measurements in overweight/obese children were significantly higher than in healthy subjects, indicating that all anthropometric measurements except for subnasal-nasion in the children with hypothyroidism or overweight/obesity were significantly different compared to the control group. Regarding non-syndromic hypogrowth, the zygomatic width was significantly lower, and the nasal base width was significantly higher than in healthy subjects.

**Table 3:** Anthropometric measurements on the study groups at all ages

Measurement	Healthy children (*n* = 587)	Medicated hypothyroidism (*n* = 216)		Overweight/obesity (*n* = 108)		Non-syndromic hypogrowth (*n* = 250)		Other disorders (*n* = 347)	
	Median ± SD mm	Median ± SD mm	*p*-value^a^	Median ± SD mm	*p*-value^a^	Median ± SD mm	*p*-value^a^	Median ± SD mm	*p*-value^a^
Head circunference	52.9 ± 2.5	53.5 ± 2.2	0.0014^a^	54.7 ± 2.0	0.000^a^	52.7 ± 2.2	0.183	53.2 ± 2.3	0.1237
Zygomatic width	110.9 ± 10.2	112.9 ± 10.9	0.011^a^	118.9 ± 13.8	0.000^a^	107.7 ± 10.7	0.0003^a^	111.7 ± 11.6	0.229
Nasal base width	28.1 ± 5.9	29.4 ± 5.8	0.003^a^	29.9 ± 6.9	0.009^a^	29.1 ± 5.8	0.016^a^	29.0 ± 6.2	0.040^a^
Intercommissural distance	42.9 ± 5.1	43.8 ± 5.3	0.0164^a^	43.7 ± 6.0	0.046^a^	43.2 ± 5.6	0.115	43.4 ± 5.8	0.127
Nasion-subnasal	55.1 ± 5.8	55.7 ± 6.8	0.115	55.4 ± 6.3	0.650	54.6 ± 6.7	0.948	54.4 ± 6.5	0.217
Subnasal-interlabial	19.6 ± 2.7	19.6 ± 2.5	0.979	20.6 ± 4.6	0.006^a^	19.5 ± 2.8	0.917	19.5 ± 2.6	0.709
Interlabial-menton	42.5 ± 5.0	42.5 ± 5.3	0.755	45.2 ± 5.5	0.000^a^	41.8 ± 5.8	0.223	42.4 ± 5.5	0.896
Cranial base length	110.6 ± 8.6	112.6 ± 9.6	0.000^a^	115.5 ± 10.0	0.000^a^	108.9 ± 10.0	0.078	110.2 ± 10.5	0.863
Maxillary length	108.4 ± 8.1	110.8 ± 8.3	0.000^a^	113.6 ± 9.1	0.000^a^	107.5 ± 11.2	0.527	108.8 ± 8.7	0.444
Mandibular length	121.6 ± 47.4	122.5 ± 10.0	0.001^a^	127.5 ± 12.5	0.000^a^	119.3 ± 10.9	0.750	120.8 ± 10.7	0.168

*n* sample size

^a^*p*-value calculated with the non-parametric Mann–Whitney *U* test

According to the Spearman correlation coefficient (rho), chronological and skeletal age were strongly and positively correlated in all groups and genders except for obese males (Table 4).

**Table 4:** Correlation between chronological and skeletal age according to disorder and gender

Disorder	Male		Female	
	rho	*p*-value	rho	*p*-value
Healthy	0.9	0.000^a^	0.8	0.000^a^
Medicated hypothyroidism	0.8	0.000^a^	0.6	0.000^a^
Overweight/obesity	0.6	0.108	0.8	0.000^a^
Non-syndromic hypogrowth	0.8	0.000^a^	0.8	0.000^a^
Others	0.8	0.000^a^	0.7	0.000^a^

^a^Spearman rho correlation coefficient

Overall, no significant differences in the mean skeletal age were observed between males and females between 5 and 15 years when the different groups of disorders were compared with the controls. However, increased skeletal age was found in healthy males between 10 and 15 years when compared to hypothyroid individuals (*p* = 0.037), in healthy females between 5 and 9 years compared to non-syndromic hypogrowth patients (*p* = 0.001), and in healthy males between 10 and 15 years compared with non-syndromic hypogrowth males (*p* = 0.001).

Considering that the Tanner II and IV indexes are related to the maximum peak of skeletal maturation in females and males, respectively, the different anthropometric measurements were compared between the groups according to these variables (Table 5). Male (Tanner IV) hypothyroid patients did not present significant differences in anthropometric measurements compared to healthy patients. In contrast, female (Tanner II) hypothyroid females showed significant differences in head circumference (*p* = 0.035) and the interlabial-menton distance (*p* = 0.030) compared to the controls. Moreover, in overweight/obese males and females, several anthropometric measurements were significantly higher than in healthy individuals. Finally, non-syndromic hypogrowth males and females showed a significantly lower zygomatic width (*p* = 0.023) and head circumference (*p* = 0.049), respectively, when compared to the controls. (Table 6).

**Table 5:** Comparison of skeletal age according to chronological age group and gender

Age group (years)	Gender	Healthy children	Medicated hypothyroidism		Overweight/obesity		Non-syndromic hypogrowth		Other disorders	
		Median ± SD	Median ± SD	*p*-value	Median ± SD	*p*-value	Median ± SD	*p*-value	Median ± SD	*p*-value
5–9	Female (*n* = 273)	7.4 ± 2.0	8.1 ± 1.9	0.29	8.1 ± 1.5	0.559	5.8 ± 2.2	0.001^a^	8.3 ± 2.2	0.017
	Male (*n* = 174)	6.3 ± 1.9	6.0 ± 1.2	0.618	8.7 ± 1.6	0.17	6.5 ± 2.0	0.628	7.0 ± 2.5	0.14
10–15	Female (*n* = 345)	10.3 ± 2.1	10.2 ± 1.9	0.977	10.3 ± 1.3	0.543	9.5 ± 2.2	0.018	10.7 ± 1.5	0.243
	Male (*n* = 366)	11.7 ± 2.0	10.1 ± 3.0	0.037^a^	10.6 ± 2.2	0.246	10.4 ± 2.5	0.001^a^	11.7 ± 1.9	0.931

*n* sample size

^a^*p*-value calculated with the non-parametric Mann–Whitney *U* test

**Table 6:** Anthropometric measurements in the different groups according to gender and Tanner II (females) and IV (males) indexes

Gender	Tanner	Measurement (mm)	Healthy children	Medicated hypothyroidism	*p*-value	Overweight/obesity	*p*-value	Non-syndromic hypogrowth	*p*-value	Other disorders	*p*-value
			Median ± SD	Median ± SD		Median ± SD		Median ± SD		Median ± SD	
F	Tanner II (*n* = 116)	Head circunference	52.7 ± 1.7	51.3 ± 2.0	0.035^a^	54.6 ± 1.2	0.002^a^	51.5 ± 1.5	0.049^a^	53.2 ± 1.8	0.264
		Zygomatic width	109.3 ± 7.8	108.2 ± 8.9	0.869	119.0 ± 7.8	0.003^a^	109.2 ± 8.1	0.920	111.8 ± 8.0	0.080
		Nasal base width	28.0 ± 5.0	31.3 ± 4.7	0.082	31.4 ± 4.8	0.051	27.8 ± 5.6	0.902	28.1 ± 5.6	0.993
		Intercommissural distance	42.1 ± 4.3	43.0 ± 4.7	0.634	42.9 ± 6.3	0.233	39.2 ± 10.2	0.484	43.6 ± 3.9	0.071
		Nasion-subnasal	54.3 ± 5.3	52.7 ± 6.0	0.289	57.5 ± 5.9	0.130	54.4 ± 4.4	0.984	55.8 ± 4.1	0.335
		Subnasal-interlabial	18.7 ± 2.2	18.7 ± 2.0	0.676	19.7 ± 3.1	0.229	19.3 ± 2.4	0.296	19.5 ± 2.4	0.119
		Interlabial-menton	41.6 ± 2.9	38.7 ± 4.0	0.030^a^	44.6 ± 2.9	0.011^a^	40.1 ± 4.2	0.339	41.9 ± 4.6	0.700
		Cranial base length	108.6 ± 7.2	109.1 ± 5.1	0.846	118.7 ± 2.6	0.000^a^	103.6 ± 11.2	0.080	108.9 ± 10.9	0.257
		Maxillary length	107 ± 5.0	10.5 ± 5.5	0.540	114.6 ± 2.5	0.000^a^	102.9 ± 6.8	0.051	107.3 ± 7.1	0.426
		Mandibular length	118.5 ± 6.1	116.7 ± 6.3	0.307	127.1 ± 2.9	0.000^a^	116.2 ± 5.7	0.258	118.4 ± 6.1	0.731
M	Tanner IV (*n* = 262)	Head circumference	54.5 ± 1.8	55.0 ± 1.5	0.108	56.0 ± 2.0	0.006^a^	54.2 ± 1.7	0.154	54.9 ± 1.7	0.148
		Zygomatic width	115.7 ± 8.6	118.2 ± 8.7	0.120	121.1 ± 19.1	0.002^a^	112.9 ± 8.0	0.023^a^	116.8 ± 15.2	0.663
		Nasal base width	29.9 ± 6.7	31.7 ± 6.3	0.164	32.2 ± 9.3	0.403	31.1 ± 6.5	0.242	33.1 ± 6.1	0.006^a^
		Intercommissural distance	47 ± 4.2	45.7 ± 6.1	0.669	49.0 ± 4.4	0.168	46.9 ± 3.4	0.908	47.4 ± 3.9	0.564
		Nasion-subnasal	58.5 ± 4.7	57.9 ± 4.7	0.557	56.5 ± 3.7	0.085	58.7 ± 4.2	0.661	58.8 ± 5.4	0.809
		Subnasal-interlabial	21.1 ± 2.5	21.6 ± 2.7	0.286	23.7 ± 9.9	0.416	21.0 ± 2.5	0.848	20.6 ± 2.7	0.239
		Interlabial-menton	45.9 ± 4.6	45.8 ± 5.2	0.735	50.3 ± 4.7	0.001^a^	45.4 ± 4.8	0.680	46.1 ± 4.9	0.535
		Cranial base length	116.9 ± 6.9	118.1 ± 7.2	0.114	118.1 ± 16.4	0.006^a^	115.5 ± 8.4	0.449	117.1 ± 9.2	0.311
		Maxillary length	116.2 ± 5.3	117.4 ± 5.9	0.147	118.0 ± 16.6	0.003^a^	116.6 ± 6.1	0.747	116.6 ± 6.9	0.550
		Mandibular length	129.1 ± 7.3	130.1 ± 7.4	0.119	134.5 ± 21.1	0.000^a^	128.7 ± 6.6	0.752	130.4 ± 9.0	0.254

*n* sample size

^a^*p*-value calculated with the non-parametric Mann–Whitney *U* test

## Discussion

This study aimed to investigate the influence of overweight, obesity, and endocrine disorders, i.e., medicated hypothyroidism and non-syndromic hypogrowth, on maxillary and mandibular growth in 1508 children and adolescents between 5 and 19 years. Overall, medicated hypothyroid and obese subjects had increased anthropometric measurements. In addition, at peak growth, obese subjects showed increased measurements, while medicated hypothyroid and non-syndromic hypogrowth patients presented decreased measurements.

Of the conditions studied, the most prevalent in children and adolescents in Colombia and other populations are overweight and obesity, followed by medicated hypothyroidism and non-syndromic hypogrowth (Minsalud 2022; Danze et al. 2021; Chaves et al. 2018). However, in this patient cohort, non-syndromic hypogrowth (16.6%) was the most frequent, followed by medicated hypothyroidism (14.3%) and overweight/obesity (7.2%). This marked difference could be explained by the fact that the studied population studied was undergoing treatment, and there is a lower proportion of obese children and adolescents that seek treatment at Pediatric Endocrinology services, where the sample was collected.

In this study, patients with such conditions presented with altered craniofacial anthropometry in the sagittal, transverse, and vertical planes compared to healthy controls. In addition, hypothyroid subjects showed increased head circumference, zygomatic width, nasal base width, intercommissural distance, cranial base length, and maxillary and mandibular length. These findings accord with those reported by Gunes et al. (2020), which suggest that hypothyroid individuals could present early changes in body composition parameters. In contrast, our results diverge from those of Vucic et al. (2017), who reported an association between hypothyroidism and decreased maxillary and mandibular growth, which could result in teeth impaction or delayed tooth eruption (Wassner 2017). However, it should be noted that these studies were conducted on non-treated individuals.

No significant differences were found in overweight/obese patients compared to the controls for the above anthropometric measurements. However, subnasal-interlabial and interlabial-menton distances were greater, giving them a leptoprosopic facial appearance. Some studies also indicate a relationship between overweight/obesity and bone growth stimulation (Dimitri 2019; Zhu 2017). Our findings on craniofacial growth at peak growth in patients with overweight/obesity are in agreement with other investigations, which reported a more sagittal position of pogonion, leading to prognathism (Danze et al. 2020), an increased longitudinal facial length (Cuccia et al. 2007), and early eruption in both dentitions (Sánchez et al. 2010). Finally, zygomatic width was significantly smaller in non-syndromic hypogrowth patients than in healthy subjects, which aligns with the findings from Attanasio and Shalet (2007) and Oliveira-Neto et al. (2011).

When anthropometric measurements at peak growth were analyzed, boys with medicated hypothyroidism had significantly lower values than healthy men, suggesting delayed linear growth and skeletal development, as reported in other studies (Williams 2018). Moreover, head circumference and interlabial-menton circumference in girls with medicated hypothyroidism were significantly lower than in healthy women, while no differences were observed in males. In contrast, overweight and obese patients showed significantly increased measurements than controls, indicating that the peak growth in these individuals obese patients occurs earlier, as has been shown previously (Danze et al. 2020). Finally, girls with non-syndromic hypogrowth presented significantly decreased head circumference, while boys showed a decreased zygomatic (facial) width.

Compared to healthy controls, patients with hypothyroidism (10–15-year-old males) and non-syndromic hypogrowth (5–9-year-old females and 10–15-year-old males) showed a significant delay in the skeletal age in comparison with chronological age.

The findings of this study were to establish craniofacial growth parameters by comparing facial anthropometric measurements of healthy children with patients that presented medicated hypothyroidism, overweight-obesity or non-syndromic hypogrowth, to understand how different growth disorders can influence craniofacial growth and development, as well as identifying the timing for maxillary orthopedic intervention depending on the type any underlying disorder, based on how it may influence the onset of peak growth.

The strengths of this study include (1) all patients were examined and diagnosed by a single professional, which reduces the risk of observer bias; (2) the examiner was trained and standardized to take anthropometric measurements; (3) the use of standard anthropometric measurements in both diseased and healthy individuals reduces the risk of analysis bias and facilitate their utilization in clinical practice; and (4) the craniofacial anthropometry was performed directly in the patients without the use of additional diagnostic aids, reducing measurement bias. Regarding its limitations, due to the COVID-19 pandemic, some patients were not appointed in person but virtually for a few months. Therefore, performing direct craniofacial anthropometry in those patients was impossible. In addition, the skeletal age of some patients was not available because carpal radiographs were not indicated in all cases. Hence, Tanner stages assessment was central in predicting skeletal maturation.

## Conclusions

Considering any limitations of the present study in Colombian children and adolescents, the following conclusions can be made:

- Several anthropometric measurements were increased in hypothyroid, overweight, and obese patients compared to healthy individuals and decreased in those with nonsyndromic hypogrowth.
- Specifically, girls with hypothyroidism or non-syndromic hypogrowth exhibited decreased head circumference and interlabial-menton distance at peak growth, while overweight/obese patients of both sexes presented increased values.
- A significantly lower zygomatic width was observed in boys with non-syndromic hypogrowth.
- These findings can be an important tool in the hands of pediatric dentists and orthodontists when diagnosing and planning maxillary and mandibular orthopedic treatment.
